# Diagnostic Latency and Unmeasured Disease Severity in Male Lupus Nephritis

**DOI:** 10.1016/j.ekir.2026.106591

**Published:** 2026-05-08

**Authors:** Parankush Upadhyay

**Affiliations:** 1 Department of Nephrology, Sanjay Gandhi Postgraduate Institute of Medical Sciences, Lucknow, Uttar Pradesh, India

To the Editor:

We read with great interest the pooled analysis by Nasr *et al.*^1^ examining sex differences in lupus nephritis using data from randomized trials and international cohorts. The authors did commendable work assembling 1 of the largest and most geographically diverse datasets addressing this long-standing question.

Few differences between males and females in presentation have been previously described by Ramírez *et al.*^2^ Although this study demonstrates broadly similar histologic class distribution, treatment responses, and outcomes between males and females, the finding of significantly lower baseline estimated glomerular filtration rate in males warrants further consideration. We suggest that this difference may reflect factors upstream of biopsy classification, particularly a lower index of clinical suspicion for systemic lupus erythematosus and lupus nephritis in males, potentially leading to delayed diagnosis and later presentation.

Importantly, recorded duration of systemic lupus erythematosus or lupus nephritis may not fully capture diagnostic latency. Early manifestations in males may be attributed to alternative diagnoses, resulting in biopsy at a more advanced stage of kidney involvement despite comparable reported disease duration. In this context, the equivalence of ISN/Renal Pathology Society class does not exclude meaningful differences in underlying histopathologic severity. As acknowledged by the authors, the absence of activity and chronicity indices, tubulointerstitial injury, and vascular lesions limits the ability to assess whether males presented with greater irreversible damage within the same histologic class.

Additional contributors may include sex-related differences in renal reserve and creatinine generation, which could influence creatinine-based estimated glomerular filtration rate at presentation, as well as a higher baseline blood pressure and vascular burden in males that may compound renal functional decline independent of immune activity. Health-seeking behavior and referral patterns may further accentuate these differences, particularly in nonprotocolized cohort settings.

Finally, the numerically higher relapse rates and divergence of relapse-free survival curves beyond 4 years, though not statistically significant, raise the possibility of limited power to detect sex-specific differences rather than true equivalence.

Taken together, these considerations suggest that similar histologic class at biopsy may not fully reflect comparable disease trajectory at presentation. Future studies incorporating standardized histopathologic indices, alternative filtration markers, and measures of diagnostic delay may better elucidate the interplay between sex, disease recognition, and renal outcomes in lupus nephritis.

## Disclosure

The author declared no competing interests.
